# Injectable cartilaginous template transformed BMSCs into vascularized bone

**DOI:** 10.1038/s41598-018-26472-8

**Published:** 2018-05-29

**Authors:** Xiaoke Feng, Zhiye Li, Jianhua Wei, Zhihong Feng, Wei Wu, Yimin Zhao

**Affiliations:** 1 State Key Laboratory of Military Stomatology & National Clinical Research Center for Oral Diseases & Shaanxi Key Laboratory of Stomatology, Department of Prosthodontics, School of Stomatology, the Fourth Military Medical University, Xi’an, Shaanxi 710032 China; 2 State Key Laboratory of Military Stomatology & National Clinical Research Center for Oral Diseases & Shaanxi Clinical Research Center for Oral Diseases, Department of Oral & Maxillofacial Surgery, School of Stomatology, the Fourth Military Medical University, Xi’an, Shaanxi 710032 China

## Abstract

Regeneration of alveolar bone for dental implant remains a major issue, partifcularly for patients suffering from severe bone adsorption and irregular socket trauma. Recapitulating embryological development is becoming an attractive approach for engineer organ or three-dimensional tissues from stem cells. In this study, we aimed to develop an injectable “cartilaginous” graft with adequate mechanical resistance and ideal bone remodelling potential. The cartilaginous graft was composed of a particulate decellularised cartilage matrix (PDCM), chondrogenically primed bone mesenchymal stem cell (BMSC) bricks (CB), and enriched platelet-rich plasma (P) gel. In immunodeficient mice, we found that angiogenesis occurred quickly inside PDCM-CB-P constructs after implantation, thereby improving tissue survival and bone formation. In rabbit tibia bone defects around implants, we confirmed that CBs not only transformed into bone tissue rapidly, but also significantly promoted bone remodelling and replacement of PDCM, thus realising osseointegration of dental implants within 3 months. In conclusion, CBs exhibited the potential for endochondral ossification *in vivo*, and application of a cartilaginous template composed of PDCM, CB, and P provided a minimally-invasive, “free material residual” approach to regenerate alveolar bone tissues *in vivo*. This method could have applications in peri-implant bone regeneration.

## Introduction

Regeneration of alveolar bone for dental implants remains a major challenge, particularly for patients suffering from severe bone adsorption and irregular socket trauma. Despite the beneficial effects of solid bioceramics such as hydroxyapatite and beta-tricalcium phosphate, novel minimally-invasive, less immune-reactive substitutes are needed for complete remodelling alternatives to current therapies.

Although decellularised and demineralised bone matrices (DBMs) are effective bone reparative materials, these materials are produced xenogenically or allogeneically, and transmission of infectious microorganisms is a possibility that compromises clinical safety. Moreover, the dense native structure of DBMs reduces the efficiency of *in vivo* remodelling, including angiogenesis^1^. Recapitulating embryological development is becoming an attractive approach for engineering organs or three-dimensional (3-D) tissues from stem cells^2^. Endochondral ossification is the process through which BMSCs aggregate and chondrogenically differentiate, resulting in the formation of a cartilage template. Angiogenic and chemotactic factors are then excreted by transformed BMSCs and further attract host cells to remodel the mineralised cartilage into vascularised bone^3^. For chondrogenic differentiation, harvesting BMSCs from bone marrow, and reconstituting them into cell macroaggregates may significantly enhance the efficiency of chondrogenic differentiation and has been widely studied for cartilage regeneration^4^. By benefitting from gap junction-mediated intercellular contacts and interactions between cell-extracellular matrices^5^, the chondrogenic differentiation of BMSCs in the aggregating model was significantly enhanced by exogenous growth factors as compared with that of solid scaffold-based cell transplants^6^. Therefore, the establishment of clinically applicable “developmental engineering” technology may provide a new approach for peri-implant bone regeneration.

The development of injectable cell macroaggregates may offer a micro-invasive and shapeable approach to alveolar bone regeneration, even for loading dental implants. For alveolar bone regeneration via injectable grafts, adequate mechanical resistance and rapid osteogenic remodelling remain challenges for peri-implant filling^7,8^. Hydrogels enforced chemically or physically are therefore developed to provide a 3-D niche with enhanced toughness for seeding cells^9,10^. However, *in vivo* behaviours remain less than satisfactory owing to inflammatory reactions, interior necrosis, and poor osseointegration^11^. Blood clot-mediated socket bone healing has great potential for host remodelling, and platelets carrying multiple factors may play important roles in vascularisation and host remodelling. Moreover BMSC-platelet-rich plasma (PRP) compounds exhibit significant bone forming potential in humans; however, contraction and intrinsic mechanical weakness still limit the applications of these materials. Attempts to reconstitute BMSC-PRP mixtures with solid bioceramics, however, show reduced remodelling potential owing to the toughness and slow biodegradation of the materials. As alternatives to cell-plasma-bioceramic mixtures, “cartilaginous” grafts showing adequate mechanical resistance may show more potential for remodelling and clinical translation.

Cartilage decellularisation using several washing steps with enzymatic agents and detergents leads to full decellularisation, although the matrix microstructure remains intact, including the original mechanical properties and some biological properties. Thus, in this study, instead of seeding BMSCs onto the decellularised cartilage extracellular matrix (ECM), we fabricated particulate decellularised cartilage, and incorporated the materials into PRP to increase the mechanical resistance. Furthermore, “cell bricks” processed from fragmentation of chondrogenically differentiated BMSC macroaggregates (hereafter referred to as “CBs”) were acquired and dispersed into the above mediator. We then examined whether the proposed “cartilaginous PRP gelling compound” could regenerate new bone through endochondral ossification around dental implants. Our results provide important insights into the applications of decellularised cartilage matrices, CBs and PRP in clinical treatments.

## Results

### Characterisation of BMSC derived chondrocyte macroaggregates, PDCMs and injectable PDCM-CB-P gel

**Figure 1:**
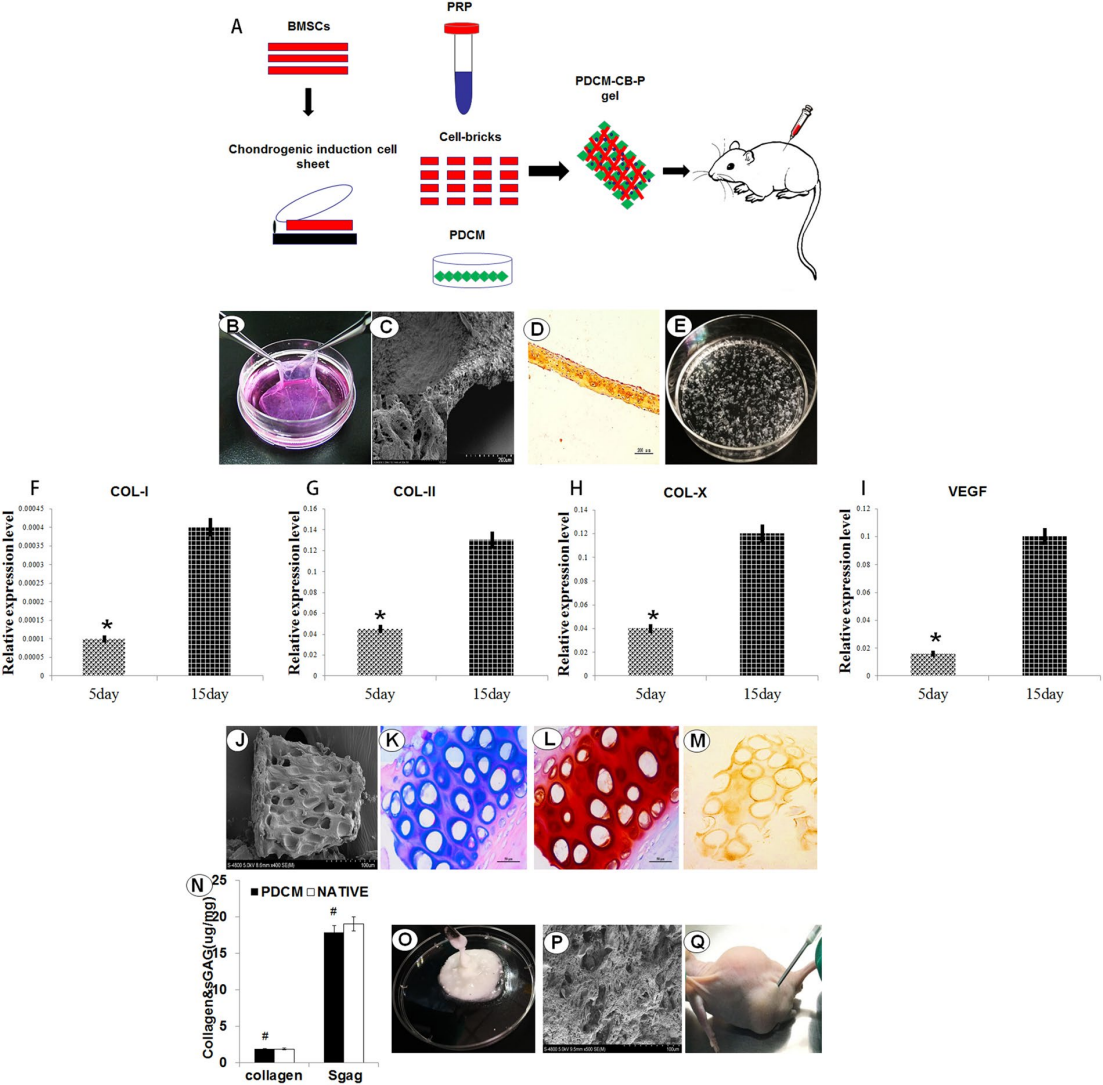
Characterisation of chondrogenic primed BMSC bricks (CBs), particulate decellularised cartilage matrices (PDCMs) and injectable PDCM-CB-P composites. (**A)** Schematic description of the strategy for cell bricks, PDCM, PRP, and *in vivo* implantation. (**B**) The chondrogenic primed BMSC sheet was cultured and harvested. (**C**) Scanning electron microscopy images showing multiple layers of cells linked by extracellular matrices; bar = 200 μm, magnification = 200× (left bottom, bar = 10 μm; magnification = 4000×). (**D**) Safranin-O staining of cell sheets after chondrogenic induction for 10 days. (**E**) Chondrogenically differentiated BMSC sheets were cut into small fragments. (**F**–**I**) Relative expression of COL-I, COL-II, COL-X, and VEGF (*P < 0.05). (**J**) Scanning electron microscopy of PDCMs, bar = 100 μm; magnification = 400×; (**K**) (**H**,**E**) staining, bar = 50 μm; magnification = 20×. (**L**,**M**) Safranin-O and immunohistochemiscal staining of COL-II, bar = 50 μm; magnification = 20×. (**N**) Quantification of collagen and sGAG. ^#^*P* > 0.05. Bar = 50 μm; magnification = 20×. (**O**) PDCM-CB-P gel. (**P**) Scanning electron microscopy of the PDCM-CB-P constructs, bar = 100 μm, magnification = 500×. (**Q**) PDCM-CB-P constructs were injected into the subcutaneous region of nude mice and formed a hemispheric graft. PDCM, particulate decellularised cartilage matrices; CB, cell bricks; P, platelet-rich plasma.

Figure 1A shows a schematic of the study design. At the end of the culture, the seeded BMSCs were chondrogenically primed to produce sufficient ECM and transformed into a semitransparent white membrane (Fig. 1B). SEM images confirmed that chondrocyte cell sheets were composed of multiple layers of cells and linked with abundant of ECM (Fig. 1C). For histological analysis of cell sheets, safranin-O staining showed that a large amount of glycosaminoglycan (GAG) occupied the extracellular space, which confirmed the formation of cartilaginous ECM (Fig. 1D). Figure 1E shows the CBs, Cell sheets were cut into small fragments, which came from the chondrogenic BMSC macroaggregates. Compared with the 5 days of cell culture, after chondrogenic induction for 10 days, the chondrocyte-specific genes COL-I, COL-X, and VEGF were significantly upregulated (Fig. 1F,H,I, *P < 0.05). Elevated expression of the above proteins in BMSCs demonstrated that BMSC macroaggregates entered pre-hypertrophy.

### Characteristics of PDCMs

As shown in Fig. 1J, the cartilage was turned into powder-like mini-fragments with a sizes ranging from 50 to 200 μm after grinding and decellularisation processes, which allowed the PDCMs to be injected. Moreover, both H&E and safranin-O (Fig. 1K,L) showed that the cells were eliminated, and the left matrices resembled a sifter. In addition, immunohistochemical staining of COL-II (Fig. 1M), safranin-O staining (Fig. 1L), and quantification of collagen and sGAG (Fig. 1N, ^#^*P* > 0.05) in PDCMs all showed that PDCMs contained the most collagen and sGAG despite undergoing grinding and decellularisation. Figure 1O shows the prepared PDCM-CB-P gel with adequate mobility. SEM images of PDCM-CB-P constructs confirmed the observation from H&E staining of PDCMs, and proved that PDCM performed as a framework within the PRP gel, forming multiple, enclosed cavities prepared for BMSC filling (Fig. 1P). Figure 1Q shows the injection process.

### Gross morphology and quantification of collagen and sGAG

**Figure 2:**
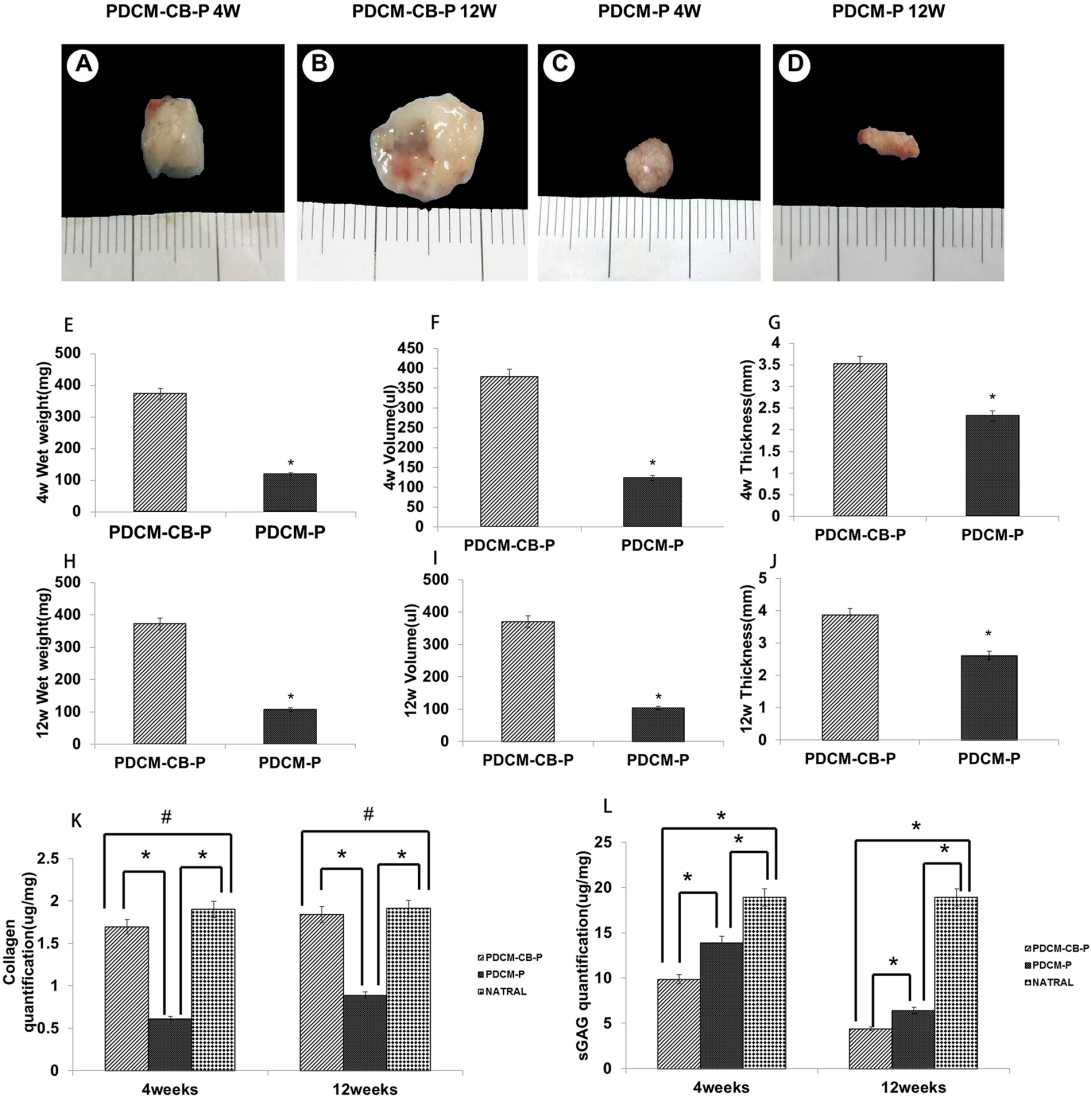
Macroscopic examination of regenerated tissues from grafts. (**A**–**D**) Close macroscopic views of the regenerated grafts from the PDCM-CB-P and PDCM-P groups at 4 and 12 weeks after *in vivo* incubation in nude mice. (**E**–**J**) Samples were harvested from nude mice at 4 and 12 weeks after operation and wet weights, volumes and thicknesses were determined. *P < 0.05. (**K**,**L**) Collagen and sGAG quantification at 4 and 12 weeks. *P < 0.05, ^#^*P* < 0.05. PDCM, particulate decellularised cartilage matrix; CB, cell brick; P, platelet-rich plasma.

At 4 weeks after injection, observable knobbles were detected in all animals. Gross morphology revealed that CB-enriched constructs (PDCM-CB-P) transformed into tissues with a white, pearly appearance (Fig. 2A), and resisted *in vivo* contraction (Fig. 2A,B) as compared with the PDCM-P group (Fig. 2C,D) which contracted significantly. After 12 weeks, the PDCM-CB-P constructs still maintained their original morphology and turned into harder tissues (Fig. 2B), whereas PDCM-P constructs exhibited a rough appearance and greater contracted (Fig. 2D). In order to quantitatively evaluate the roles of CBs played in the resistance of graft shrinkage and deformation, we measured the wet weights, volumes, and thicknesses of regenerated tissues at 4 and 12 weeks after operation. As shown in Fig. 2E,H, wet weights differed significantly among groups (4 weeks: n = 6, *t* = 6.187, *P* < 0.001; 12 weeks: n = 6, *t* = 5.813, *P* < 0.001). After 4 weeks, the wet weight of the PDCM-CB-P group (372.2 ± 43.6 mg) was significantly larger than that of the PDCM-P group (118.8 ± 18.7 mg, *P* < 0.001). After 12 weeks, the wet weight of the PDCM-CB-P group (371.5 ± 40.8 mg) was significantly larger than that of PDCM-P group (106.8 ± 10.7 mg, *P* < 0.01). Accordingly, volume measurements also indicated significant differences among the groups (4 weeks: n = 6, *t* = 6.412, *P* < 0.001; 12 weeks: n = 6, *t* = 5.795, *P* < 0.001). As shown in Fig. 2F, the volume of the PDCM-CB-P group (378.7 ± 17.6 μL) was significantly higher than that of the PDCM-P group (123.5 ± 17.8 μL, *P* < 0.01). After 12 weeks (Fig. 1I), the volume of the PDCM-CB-P group (369.3 ± 25.8 μL) was significantly higher than that of the PDCM-P group (108.8 ± 12.5 μL, *P* < 0.01). Moreover, the thickness of the samples was significantly different after 4 (n = 6, *t* = 3.411, *P* < 0.05) and 12 weeks (n = 6, *t* = 4.970, *P* < 0.001). At 4 weeks, the thickness of the PDCM-CB-P group (3.5 ± 0.9 mm) was significantly higher than those of the PDCM-P (2.3 ± 0.5 mm, *P* < 0.05) group (Fig. 2G). At 12 weeks (Fig. 2J), the thickness of the PDCM-CB-P group (3.8 ± 0.2 mm) was significantly higher than that of the PDCM-P (2.6 ± 0.4 mm, *P* < 0.001). Quantification of collagen and GAG within samples revealed significant differences among groups (4 weeks, n = 6, collagen, *F* = 663.328, *P* < 0.001; sGAG, *F* = 209.761, *P* < 0.001; 12 weeks, n = 6, collagen *F* = 389.116, *P* < 0.001; sGAG, *F* = 1260.9, *P* < 0.001). After 4 weeks, the PDCM-CB-P group exhibited the higher collagens levels (1.80 ± 0.53 μg/mg) than the PDCM-P group (0.88 ± 0.06 μg/mg), although slightly lower than that in native cartilage (1.91 ± 0.10 μg/mg; ^#^*P* > 0.05). However, sGAG (9.88 ± 0.70 μg/mg) was lower than that in native cartilage (18.91 ± 0.12 μg/mg, *P* < 0.05) and that in the PDCM-P group (13.91 ± 0.86 μg/mg, *P* < 0.05). After 12 weeks, the PDCM-CB-P group exhibited the highest amount of collagen and relatively low sGAG content (1.85 ± 0.11 μg/mg; sGAG, 4.39 ± 1.13 μg/mg). The collagen content was only slightly lower than that in native cartilage (1.9 ± 0.20 μg/mg, *P* > 0.05); whereas the sGAG content (18.89 ± 1.31 μg/mg, *P* < 0.05) was significantly higher than that in the PDCM-P group (collagen, 0.61 ± 0.02 μg/mg; sGAG, 6.43 ± 0.82 μg/mg, *P* < 0.05; Fig. 2K,L).The above results suggested that CBs played an important role in the maintenance of morphology and volume support.

### CB guided persistent ectopic endochondral ossification in nude mice

**Figure 3:**
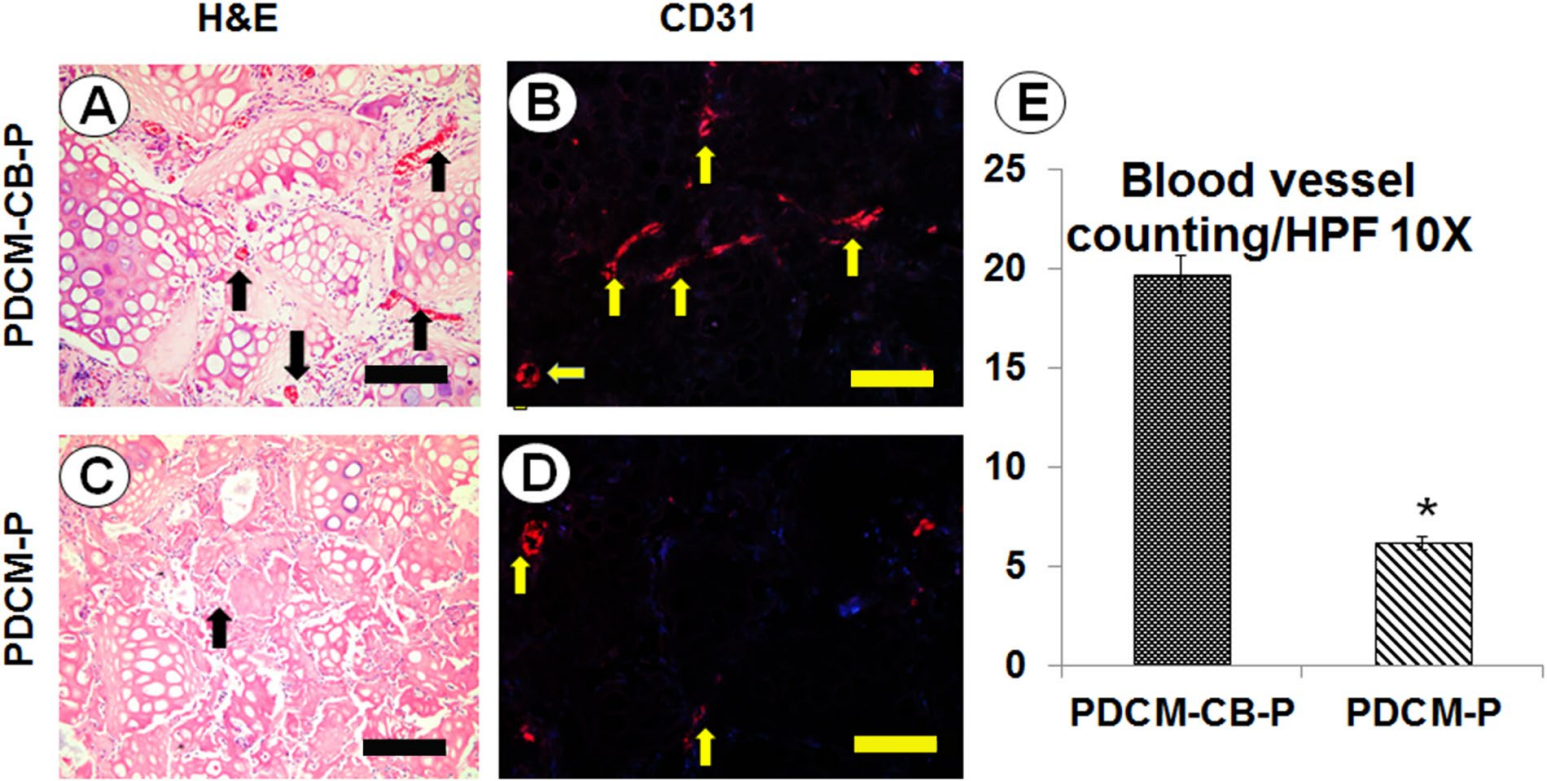
Angiogenesis regulated by cell bricks prevented central necrosis. Evaluation of angiogenesis in PDCM-CB-P constructs at week 4. (**A**,**C**) Histological images of infiltration of the capillaries at week 4, bar = 200 m, magnification = 20×. (**B**,**D**) CD31 immunofluorescent staining, bar = 200 μm, magnification = 20×. (**E**) Comparison of the number of blood vessels between the PDCM-CB-P and PDCM-P groups. The valueS represent the cumulative number of all slides examined (n = 6). *P < 0.05. PDCM, particulate decellularised cartilage matrix; CB, cell brick; P, platelet-rich plasma, HFP, high-power field.

Histological examination revealed that CB-enriched PRP gel mixed with PDCMs resisted graft contraction. Surprisingly, PDCM-CB-P constructs exhibited good tissue formation, but no central necrosis. In the PDCM-CB-P group, BMSCs maintained cell morphology and exhibited progressive angiogenesis (Fig. 3A). Higher magnification images demonstrated that circular, capillary-like tissues formed in BMSC regions of grafts as early as week 4 (Fig. 3A, black arrow). CD31 immunostaining (Fig. 3B) confirmed that the structure was comprised of endothelial cells, thus proving that BMSC regions permitted early vascular infiltration and nourished central tissues.

We then compared the numbers of blood vessels at week 4 between the PDCM-CB-P and PDCM-P groups by counting CD31-positive blood vessels. More blood vessels presented in the PDCM-CB-P group than in the PDCM-P group (Fig. 3E, *P < 0.05). These data showed that the BMSC derived chondrocyte bricks promoted early vascularisation of grafts and prevent central necrosis.

### CB guided endochondral ossification

**Figure 4:**
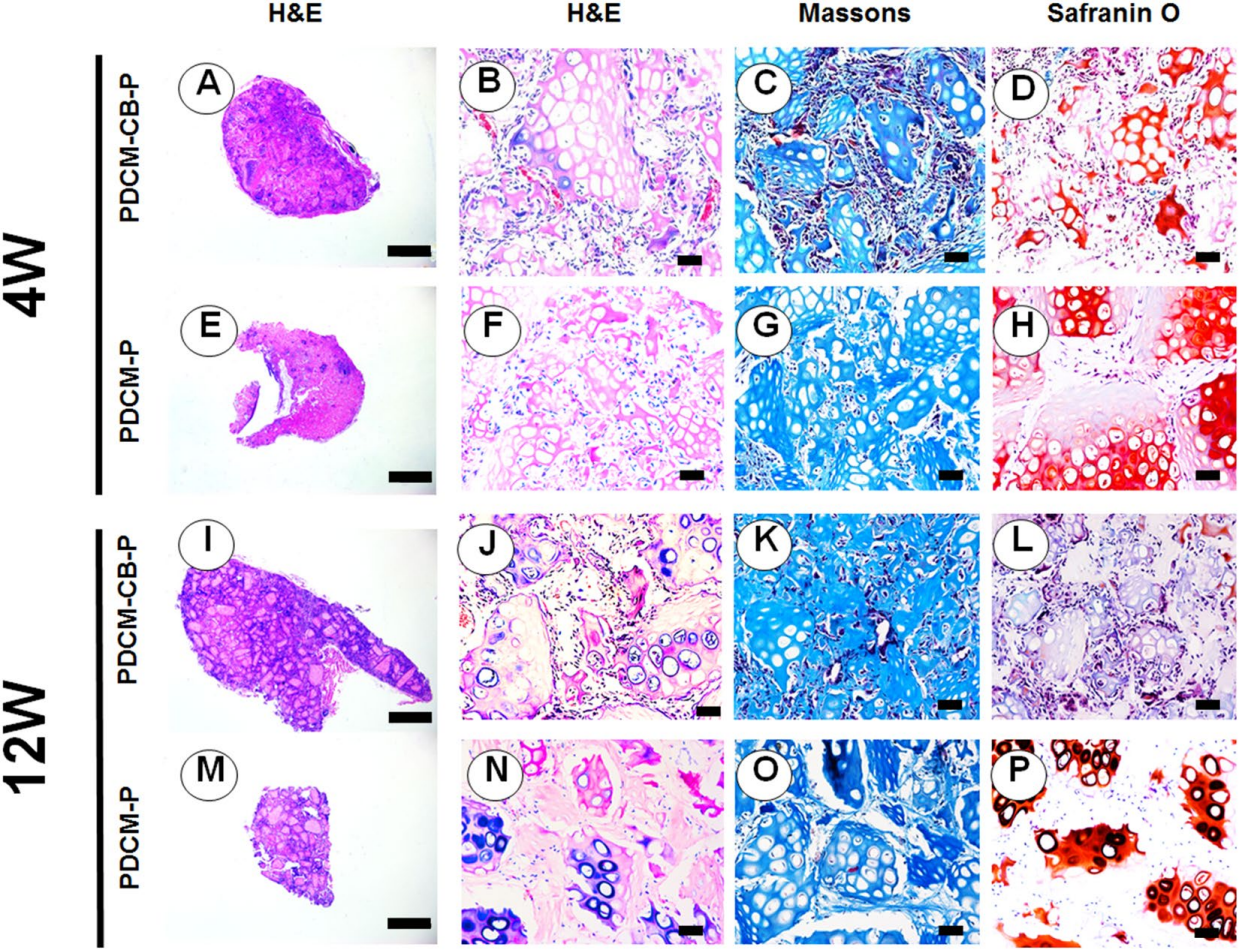
PDCM-CB-P guided osteogenesis in nude mice. Examination of the osteogenesis of grafts *in vivo* at 4 weeks (**A**–**H**) and 12 weeks (**I**–**P**) by H&E staining, Masson trichrome staining, and safranin-O staining. (**A**,**E**,**I**,**M**) Merged images showing that the PDCM-CB-P constructs acquired better vascularisation and more bone-like matrices throughout the culture. Bar = 1 mm, magnification = 1×. (**B**–**D**,**F**–**H**,**J**–**L**,**N**–**P**) **H**&**E** staining, Safranin-O staining and Masson trichrome staining of PDCM-CB-P constructs, bar = 50 μm, magnification = 20×. PDCM, particulate decellularised cartilage matrix; CB, cell brick; P, platelet-rich plasma.

The osteogenic characteristics of regenerated tissue were examined by H&E staining, Masson trichrome staining, and safranin-O staining, which further revealed different remodelling processes *in vivo* and different histological structures of engineered tissues. Following 4 weeks *in vivo*, in the PDCM-CB-P group, BMSCs developed into hard tissues (Fig. 4A), and after 12 weeks, these samples were turned into bone-like tissues (Fig. 4I).

We then used PDCM-P as a control group for evaluation of long-term endochondral ossification (Fig. 4E,M). Samples from the PDCM-CB-P group exhibited tissue formation throughout the grafts and vascular invasion into PDCM (Fig. 4B,C). In contrast, the PDCM-P group showed central necrosis with few blood vessels and mesenchyme (Fig. 4F). Masson trichrome staining and Safranin-O staining showed that the PDCM-CB-P group mineralised faster and maintained less sGAG than the PDCM-P group (Fig. 4C,D,G,H). Furthermore, 12 weeks of *in vivo* incubation resulted in mature bone-like matrices formed from CBs (Fig. 4J,K,N,O), and that the PDCM-CB-P group consumed more sGAG (Fig. 4L,P).

**Figure 5:**
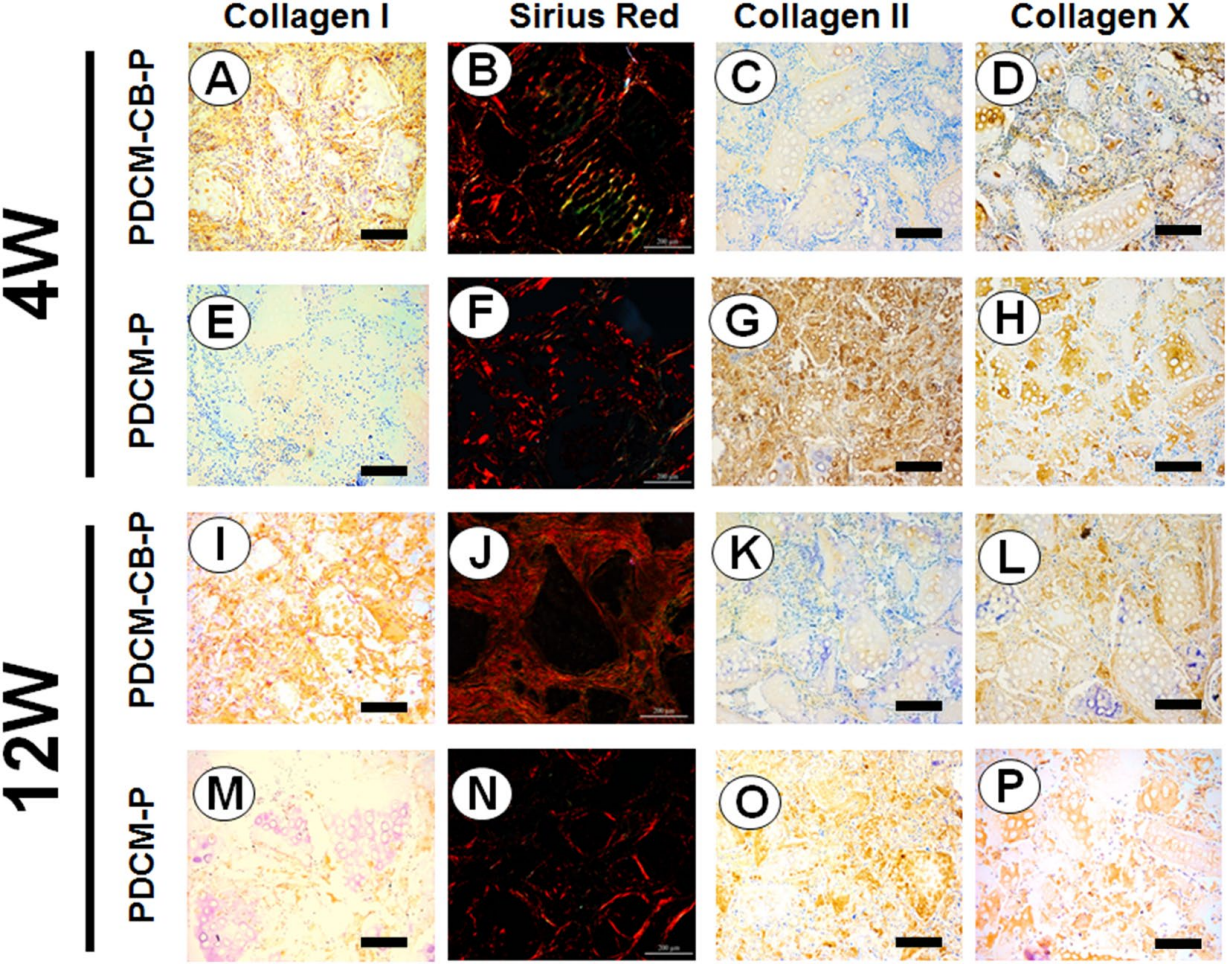
Cell bricks guided persistent deposition and production of bony collagen. Examination of the osteogenesis of grafts *in vivo* at 4 weeks (**A**–**H**) and 12 weeks (**I**–**P**). (**A**,**C**,**D**,**I**,**K**,**L**) The PDCM-CB-P constructs exhibited ossification through 12 weeks, as confirmed by the high expression of bony collagen, such as COL-I and COL-X, and the lower expressed COL-II in immunostaining. (**B**,**J**) Sirius red staining revealed that COL-II was replaced by COL-I and COL-X. (**E**–**H**,**M**–**P**) The PDCM-P constructs exhibited slower ossification than the PDCM-CB-P groups, bar = 200 μm, magnification = 10×. PDCM, particulate decellularised cartilage matrix; CB, cell brick; P, platelet-rich plasma; COL, collagen.

As indicated in Fig. 5E,G,H, we aimed to visualise ossification. Sirius red staining revealed that the graft was in the process of endochondral ossification, and COL-II (Fig. 5B, green) was replaced with COL-I and COL-X (Fig. 5B, yellow), and formed a compact collagenous fiber mesh (Fig. 5B). These findings indicated that bony collagens such as COL-I and COL-X, were abundantly expressed in the PDCM-CB-P group at 4 weeks. However, in the PDCM-P group, this progress was slower (Fig. 5F). To further identify osteogenesis in implanted CBs, we performed immunostaining for collagen types I and X. In accordance with the histological examination, BMSCs in the PDCM-CB-P group began to express collagen types I and X around PDCMs (Fig. 5A,D) at week 4, and the newly formed ECM exhibited deeper staining for the above proteins through 12 weeks (Fig. 5I,L), indicating that constructs underwent progressively osteogenesis. In contrast, in the PDCM-P group, low-level staining of collagen types I and X (Fig. 5E,H), even after 12 weeks *in vivo*; staining for both collagens was significantly weeker than those in PDCM-CB-P samples (Fig. 5M,P). In contrast, COL-II showed reduced expression in the PDCM-CB-P group compared with that in the PDCM-P group, regardless of the time after injection (Fig. 5C,K,G,O).

### Bone defect repair in the rabbit tibia model

**Figure 6:**
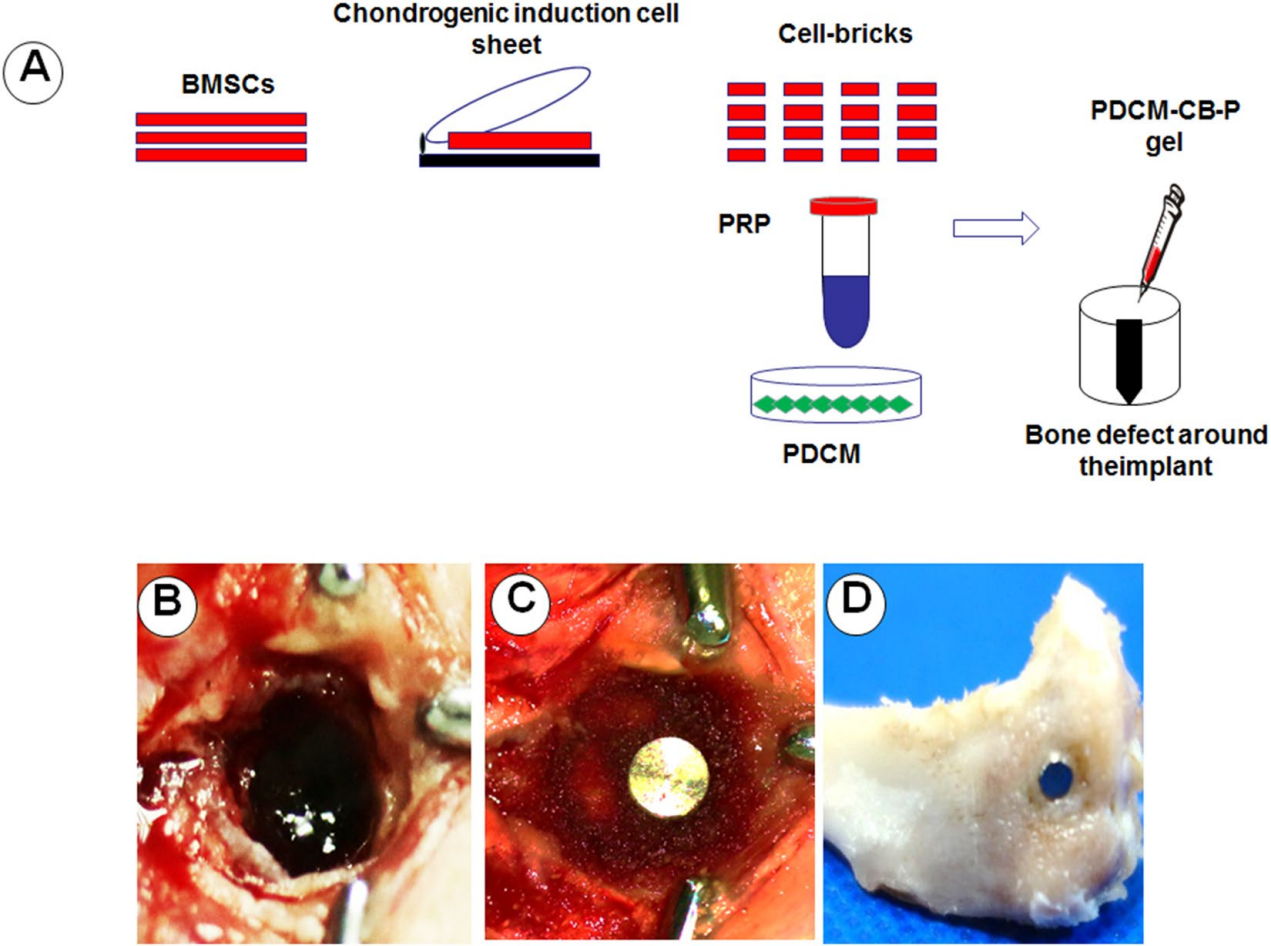
Operation in a rabbit tibia model. (**A**) Schematic description of the strategy for peri-implant bone defect repair with PDCM-CB-P constructs and other constructs. (**B**) Prepared bone defects in the rabbit tibia. (**C**) The implant was placed into the defect, and the bone defect was filled around the implant with the prepared PDCM-CB-P constructs and other constructs. (**D**) The bone grafts were the bone samples after 12 weeks. PDCM, particulate decellularised cartilage matrix; CB, cell brick; P, platelet-rich plasma.

To further examine the bone regeneration ability of PDCM-CB-P constructs, we created bone defects around implants in the tibias of New Zealand white rabbits (Fig. 6A). The gel-like construct was then injected into the prepared defects (Fig. 6B,C). After 12 weeks, the rabbits were sacrificed, and bone samples were harvested (Fig. 6D).

**Figure 7:**
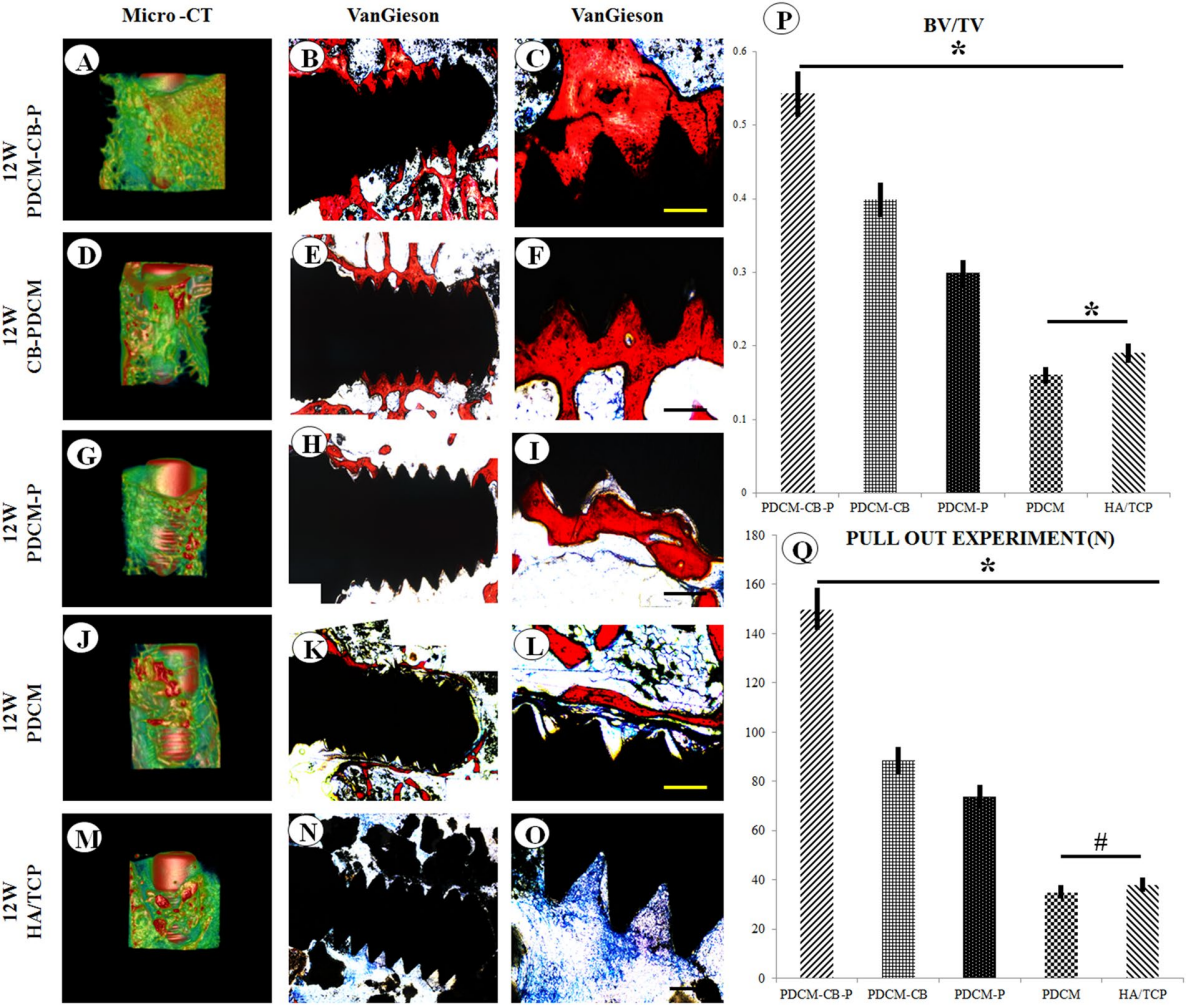
Osseointegration and mechanical characteristics of PDCM-CB-P grafts in the rabbit model after 12 weeks. (**A**,**D**,**G**,**J**,**M**) Micro-CT scanning of bone formation around the implant after 12 weeks *in vivo*. (**B**,**F**,**H**,**K**,**N**) Merged images of Van Gieson(VG) staining of samples with implants for all groups. Bone is indicated by red, and the implant indicated by black (**C**,**F**,**I**,**L**,**O**). Bar = 200 μm, magnification = 10×. (**P**) Analysis of bone volume to total volume (BV/TV). *P < 0.05. (**Q**) The pull-out experiment for analysis of mechanical properties. *P < 0.05, ^#^*P* > 0.05. PDCM, particulate decellularised cartilage matrix; CB, cell brick; P, platelet-rich plasma.

Micro-computed tomography (micro-CT) showed that significantly more bone formed around the implant in the PDCM-CB-P group than in the other groups (Fig. 7A,D,G,J,M), among which the CB-PDCM group formed more bone than the PDCM-P group, and the PDCM-P group formed more bone than the PDCM group, in the hydroxyapatite tricalcium phosphate (HA/TCP) group, very few new bone formed and the fibrous tissues grew into the empty between HA/TCP grafts and implant. The VG staining showed more bone around the implant and better osseointegration in the PDCM-CB-P group than in the other groups (Fig. 7B,F,H,K,N), among which the CB-PDCM group formed more bone than the CB-PDCM group and the PDCM-P group formed more than the PDCM group, these findings were consistent with the observations from micro-CT imaging (Fig. 7B,F,H,K,N). Furthermore, analysis of the bone volume to total tissue volume (BV/TV) showed that the PDCM-CB-P group formed more bone than the other groups (Fig. 7P); the CB-PDCM group formed more bone than the PDCM-P group and the PDCM-P group formed bone more than the PDCM group and the HA/TCP group (Fig. 7P, *P < 0.05). The pull-out experiment indicated that implants in the PDCM-CB-P group exhibited much stronger retention than those in the other groups (Fig. 7Q). For example, the CB-PDCM group showed stronger retention than the PDCM-P group and the PDCM-P group showed stronger retention than the PDCM group and the HA/TCP group (Fig. 7Q, *P < 0.05, ^#^*P* > 0.05).

**Figure 8:**
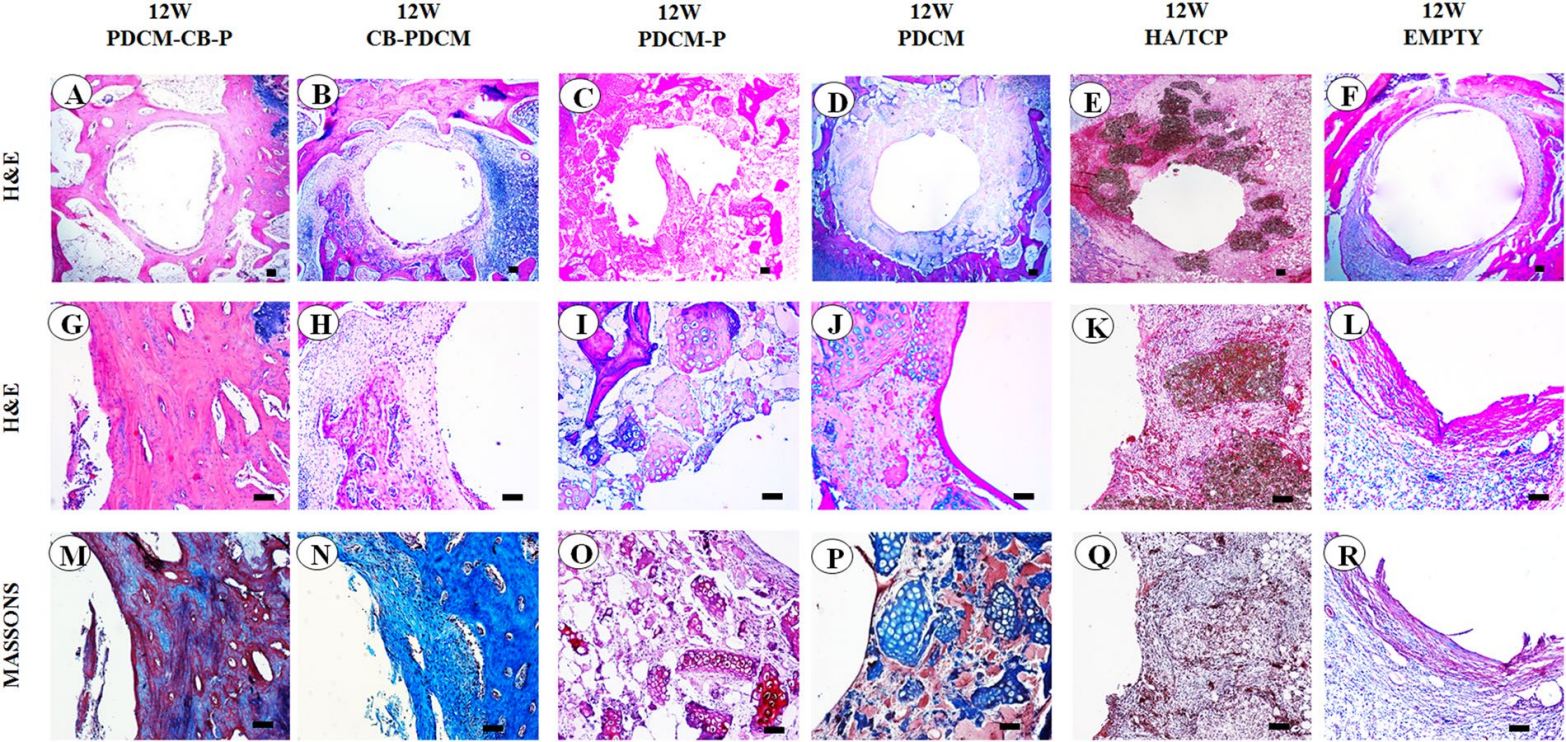
PDCM-CB-P grafts enabled stable peri-implant bone regeneration in rabbit tibias. (**A**–**F**) Merged images of all test groups showed obvious differences in bone formation. Bar = 1 mm, magnification = 4×. (**G**,**M**) Bone regeneration around the implant, as examined by histological analyses, including (**H**,**E**), and Masson’s trichrome staining. Bar = 200 μm, magnification = 10×. (**H**–**L**, **N**–**R**) Similar staining showing much less cancellous bone formation and clear PDCM or fibrous tissues remaining. Bar = 200 μm, magnification = 10×. PDCM, particulate decellularised cartilage matrix; CB, cell brick; P, platelet-rich plasm.

Histological examination further revealed the osteogenic performance of the constructs around the implants. PDCM-CB-P constructs almost completely transformed into vascularised cancellous bone in rabbits after 12 weeks (Fig. 8A,G), whereas the other groups formed varied amounts of fibrous tissues (Fig. 8B–L). The group with a hole alone showed that the defect could not repair itself and was instead, filled with fibrous tissues (Fig. 8F). CB-PDCM constructs were replaced by cancellous bone, although the amount was still less than the PDCM-CB-P constructs (Fig. 8B,H). PDCM-P constructs presented as acellular constructs *in vivo* (Fig. 8C,I), which indicated that the remodelling process was significantly different from that of the PDCM-CB-P group. The PDCM and the HA/TCP group formed the least cancellous bone (Fig. 8D,J,E,K). Consistence with subcutaneous osteogenesis in nude mice, PDCM-CB-P constructs underwent rapid bone remodelling in the tibia. Furthermore, the PDCM was completely degraded and was replaced by cancellous bone, which could contribute to the stronger remodelling capacity of rabbits compared with nude mice. Additionally, more host derived osteogenic cells were observed in the thecancellous bone environment (Fig. 8A,G). Masson trichrome staining results were consistent with the results of H&E staining, demonstrating that the PDCM-CB-P group formed more cancellous bone than any other group (Fig. 8M–R). These results demonstrated that the PDCM-CB-P gel could be adapted into a customised morphology during the injection process and later *in vivo* remodelling, thereby supporting the regeneration of bone in peri-implant regions.

## Discussion

The development of an injectable approach for bone regeneration is necessary to achieve micro-invasive surgery^12^. In contrast to other solid scaffold-based tissue engineering methods, injectable constructs require fluidity of components and instant establishment of a 3-D niche for BMSCs, which is challenging for scaffold design. We demonstrated that injectable PDCMs retained the most collagen and sGAG and possessed adequate mechanical strength; thus, these materials could stabilise the BMSCs and PRP gel efficiently once injected *in vivo*. Additional *in vivo* results revealed that the PDCMs in constructs offered adequate intrinsic resistance to surrounding pressure, and PDCM-CB-P grafts enabled morphological maintenance in a subcutaneous environment, as well as in rabbit tibias during implantation, thus providing a novel and efficient strategy for cell transplantation. These findings highlight the importance of the combination of PDCMs and CBs to establish injectable constructs for endochondral ossification *in vivo*.

The most important finding in this study is that BMSC bricks embedded in decellularised cartilage matrices in PRP gel survived well and underwent hypertrophic translation and endochondral ossification, which further indicated that such a biological graft could meet the requirements for bone augmentation by stimulating the bone formation around implant. In contrast to conventional “osteogenic grafts”, cartilage rudiment fabricated from PDCM, BMSC bricks, and PRP gel underwent rapid host remodelling *in vivo*^13^, which endowed them with more resistance to malnutrition. Moreover, BMSCs, especially those transforming into hypertrophic chondrocytes, convey essential angiogenic signals, including VEGF, matrix metalloproteinase (MMP) 9, stromal cell-derived factor 1, and granulocyte macrophage colony-stimulating factor^14–16^. Among these factors, VEGF is the most important mediator of angiogenesis, functioning to couple recruitment of endothelial cells, capillary infiltration, and ossification of hypertrophic cartilage^17–19^. Vascularisation, the process of capillary ingrowth and sprouting into the grafts, is the key step during endochondral ossification. Owing to the dense microstructure of cartilage ECM, however, seeding BMSCs on it may not result in sufficient vascularisation and tissue ingrowth. PDCM dispersed in PRP gel may leave more space or channels for invading cells, including pioneering vascular cells.

In our rabbit model, PDCM disappeared and was replaced by autologous cancellous bone in 3 months, which illustrated that the degradation process of cartilaginous ECM was well coordinated with ossification and provided sufficient bone forming space. Interestingly the main components of the injectable construct, i.e., sGAG and collagen, provided mechanical support during the coupled degradation of PDCM during osteogenesis. Our study revealed that the BMSC bricks in the PDCM-CB-P constructs resulted in faster degradation of decellularised cartilage matrices both in nude mice and in the rabbit model, but the most HA/TCP were still remained and led to few new bone formed. BMSC bricks underwent endochondral ossification after implantation and released cartilaginous matrix resolving factors, such as MMP-13^20^, which enhanced the degradation of neighboring PDCM. We also observed that PDCM degraded completely in the rabbit tibia model within 3 months, but remained intact in nude mice. We assumed that this difference may be related to inflammatory reactions and the osteogenic environment in the rabbit model. Vascular invasion and osteogenic remodelling elicited by BMSC bricks connected grafts with the surrounding host cancellous bone, and macrophages, primarily M2 macrophages, migrated from blood vessels to actively join the remodelling of PDCM^21^; this would initiate the constructive remodelling from surrounding tissue-neighbouring cancellous bone^22^. However, small amounts of bone were found to grow, indicating that host bone, in addition to BMSC bricks, also contributed to bone regeneration in PDCM-CB-PRP constructs. We also found that osseointegration occurred between the PDCM-CB-PRP and implant, revealing the high quality of newly formed bony ECM to meet functional requirements. Additionally, Sirius red staining in samples from nude mice showed increasing amounts of collagen type I among the PDCM and regularly aligned collagen fibrils, indicating the strong matrix forming capacity of BMSC derived chondrocyte bricks. Moreover, osteogenesis of constructs in bone defects indicated that host remodelling contributed to matrix formation and thus highlighting the bone remodelling potential of PDCM-CB-P constructs.

With the paradigm transition from cell transplantation to endogenous regeneration, scaffolds that enhance host cell infiltration and benefit host remodelling are thought to be more beneficial for bone regeneration^23^. Decellularised cartilage, simulating an intermediate template of endochondral ossification, was shown to induce endogenous vascularisation and osteogenesis. Decellularised hypertrophic cartilage engineered from BMSCs or mature chondrocytes undergoing hypertrophic priming have been proven to be osteoinductive *in vivo*^24^. As compared with a previous study, the cartilage we chose for our study was abundantly available, and the frozen grinding process fragmented the samples to make them easy to be decellularise. However, our study failed to show that decellularised cartilage could regenerate bone tissues without BMSC growth, neither subcutaneously nor in the tibia; this could be related to the poor endogenous osteogenic potential of mature cartilaginous ECM or destruction of biological cues caused by chemical detergents. Interestingly, integration of CBs sufficiently induced vascular invasion in PDCM-CB-P constructs and initiated bone remodelling when orthotopically implanted. This finding indicated that CBs not only offered exogenous osteogenesis but also released essential osteogenic signals to mediated host remodelling on decellularised cartilage to initiate bone remodelling. Further studies are needed to determine the exact roles and molecular mechanisms of CBs.

## Methods

### Cell isolation and fabrication of BMSC derived chondrocyte macroaggregates

All experimental protocols involving animal cells and animal experiments were approved by the Ethics Committee of the Fourth Military Medical University. All methods were performed in accordance with the relevant guidelines and regulations. Bone marrow was flushed out of the iliac cancellous bone of rabbit, dispersed with 40 mL Dulbecco’s modified Eagle’s medium (DMEM; Hyclone, Logan City, UT, USA), centrifuged at 1,000 rpm for 5 min, and then resuspended in DMEM (low glucose) supplemented with 10% foetal bovine serum (FBS; Hyclone), 50 μg/mL penicillin and 30 μg/mL streptomycin (Amresco, Cleveland, OH, USA). Primary cells were seeded in a 75 cm^2^ culture flask in DMEM (high glucose; Hyclone) containing 10% FBS and incubated at 37 °C with 5% CO_2_. The cells were digested with 0.25% trypsin (Hyclone) when cell clones reached over 80% confluence and subcultured at 1.0 × 10^4^ cells/cm^2^. Cells were then cultured in DMEM (low glucose) supplemented with 10% FBS (Hyclone), L-glutamine (272 μg/mL; Amresco), ascorbate-2-phosphate (50 μg/mL; Sigma, St. Louis, MO, USA), 50 μg/mL penicillin and 30 μg/mL streptomycin (Amresco) and incubated at 37 °C with 5% CO_2_ until the adherent cells reached 80% confluence. The medium was then changed to chondrogenic medium with high-glucose DMEM containing 50 mg/mL of gentamicin and 1.5 mg/mL fungizone (Invitrogen, Carlsbad, CA, USA), l-ascorbic acid 2-phosphate, 100 mM sodium pyruvate (Sigma), 1:100 insulin transferrin selenium (BD Biosciences, USA), 10 ng/mL transforming growth factor-β (R&D Systems, USA), and 100 nM of dexamethasone (Sigma, USA). The medium was changed every 3 days. After 10 days of culture, a thin macroaggregate formed. The obtained macroaggregate was cut into small fragments, and then suspended in serum-free DMEM. For further examination, some macroaggregates were histologically stained with safranin-O staining, subjuected to scanning electron microscopy (SEM), and examined by polymerase chain reaction (PCR) for collagen I, collagen II, collagen X, and vascular endothelial growth factor (VEGF).

### Preparation of particulate decellularised cartilage matrices (PDCMs)

PDCMs were made from rabbit ear. Cartilages were mechanically ground with a tissue grinding apparatus (TL2020, China) in liquid nitrogen for 10 min. After grinding, the cartilages were washed in phosphate-buffered saline (PBS). Furthermore, cartilages were immersed in 1% sodium dodecyl sulphate (SDS) for 96 h, and SDS was then changed every 24 h. After 96 h, cartilages were rinsed with PBS and 1% Triton X-100 for 24 h. Finally, decellularised cartilages were rinsed with nuclease for 4 h, washed with PBS, and subjected to freezing and thawing cycles for 24 h. For further examination, PDCMs were analysed by hematoxylin and eosin (H&E) staining, safranin-O staining, immunohistochemical staining for collagen II, SEM, and quantification of collagen and sulphated glycosaminoglycans (sGAGs).

### Preparation of PRP and PDCM-CB-P and PDCM-P constructs

Whole blood was aspirated from the ventricles of rabbits and mixed with sodium citrate (3.8%) at a ratio of 9:1 for anti-coagulation. PRP was enriched through a two-step centrifugation process. Briefly, 9 mL whole blood containing 1 mL sodium citrate (3.8%) solution were spun at 2,000 rpm for 8 min in a centrifuge at room temperature, and the blood separated into three phases. The top and middle layers were transferred to new tubes and centrifuged again at 3,300 rpm for 8 min. The supernatant plasma was discarded, and the remaining 2 mL plasma containing precipitated platelets was blended evenly and designated PRP. PRP was preserved on ice for further steps.

A sample of 500 μL PRP was used for each construct. CBs and PDCMs were collected. For the PDCM-CB-P group, CBs comprised of BMSCs (7.5 × 10^6^ cells) and 1 mL PDCMs were centrifuged into a mixing pellet and resuspended with PRP. For the PDCM-P group, 1 mL decellularised cartilage matrix resuspended with PRP. We prepared some PDCM-CB-P gel for injection property test and mechanical test, the detailed methods and results are described in the supplementary files.

### Biochemical analysis (collagen and sGAGs content)

sGAGs were evaluated using a Blyscan™ sGAG Assay Kit (B1000; Biocolor, Carrickfergus, UK). sGAGs were extracted from specimens by digesting with papain extraction reagent (Sigma) at 65 °C for 24 h. The sGAG concentration in the supernatant was measured as the sGAG per wet weight construct from the sGAG standard curve. Collagen content was quantified using a Sircol™ Collagen Assay (S1000; Biocolor). Collagen was extracted from specimens by digesting with pepsin (1 mg/mL suspended in 0.5 M acetic acid; Santa Ana, CA, USA). Total collagen per construct wet weight was calculated from the collagen standard curve.

### Histological and immunohistochemical assays

Samples from nude mice and rabbits were fixed in 4% paraformaldehyde, embedded in paraffin, and then cut into 8-μm-thick sections. Sections were stained with H&E, safranin-O, and Masson trichrome staining. To investigate the expression levels of collagen types I, II and X in matrices, some sections were processed for immunohistochemical staining and Sirius red staining, as described previously. Briefly, the expression levels of collagen types I, II, and X were detected using primary anti-collagen type I antibodies (mouse anti-rabbit, 1:50; Abcam, Cambridge, MA USA), anti-collagen type II antibodies (mouse anti-rabbit, 1:50; Acris, Herford Germany), and anti-collagen type X antibodies(mouse anti-rabbit, 1:50; Abcam), followed by horseradish peroxidase-conjugated anti-mouse antibodies (1:200 in PBS; Santa Cruz Biotechnolohy Dallas, TX, USA), and color development was carried out using diaminobenzidine tetrahydrochloride (Santa Cruz Biotechnolohy). Some bone samples with implants from rabbits were fixed in 4% paraformaldehyde overnight, embedded in polymethylmethacrylate and then cut into 500-μm-thick sections. Sections were stained with Van Gieson (VG) to investigate the osseointegration of implants and the injected mixture.

### Immunofluorescence assay

After 4 weeks, samples from nude mice were harvested, fixed in 4% paraformaldehyde overnight, and made into frozen sections. The sections were permeabilised with 0.3% Triton X-100 at room temperature for 20 min. After being rinsed five times in PBS for 15 min, the samples were blocked with 5% goat serum for 45 min at room temperature. The samples were then incubated with primary anti-CD31 antibodies (mouse anti-rabbit, 1:100; Abcam) was incubated at 4 °C overnight, followed by Cy3-AffiniPure conjugated secondary antibodies (goat anti-mouse, 1:100; Jackson America).

### RNA isolation and real-time recerse transcription (RT)-PCR

Total RNA from CBs was extracted using RNAiso Plus (TaKaRa, Shiga, Japan) followed by a one-step phenol chloroformisoamyl alcohol extraction, as described by the manufacturer’s protocol. Real-time RT-PCR analysis of five genes, i.e., VEGF, collagen (COL-I, COL-II, COL-X), and GAPDH, was performed using a One Step SYBR PrimeScript™ RT-PCR Kit (TaKaRa). The primers used in this study were as follows: COL-I, 5′-GACATGTTCAGCTTTGTGGACCTC-3′ (sense) and 5′-GGGACCCTTAGGCCATTGTGTA-3′ (antisense); COL-II, 5′-GACCCCATGCAGTACATG-3′ (sense) and 5′-GACGGTCTTGCCCCACTT-3′ (antisense); COL-X, 5′-GGGATGCCTCTTGTCAGTGC-3′ (sense) and 5′-ATCTTGGGTCATAGTGCTGCTG-3′ (antisense); VEGF, 5′-ATCGAGACCTTGGTGGAC-3′ (sense) and 5′-CCTGGTGAGGTTTGATCC-3′ (antisense); GAPDH, 5′-TGGTATCGTGGAAGGACTCATGAC-3′ (sense) and 5′-ATGCCAGTGACGTTCCCGTTCAGC-3′ (antisense). Real-time RT-PCR was performed with five replicates. The results are presented as target gene expression first normalised to GAPDH in the same sample (ΔCt), and then to the expression of the target gene measured in chondrogenic BMSC macroaggregates as native control (ΔΔCt). The 2-ΔΔCt method was used to compare differences in gene before and after chondrogenic induction of BMSCs.

### Nude mice subcutaneous injection

Twenty-four nude mice were used for the experiment. Mice were randomly divided into two groups: the PDCM-CB-PRP (PDCM-CB-P) group, and the PDCM-PRP (PDCM-P) group. The mice were sacrificed to harvest samples at 4 and 12 weeks. The injecting process was performed as follows. First, PDCM-CB-P and PDCM-P components were aspirated into 2 mL syringes. The syringe was then aspirated with 50 μL thrombogenic agent (100 U/mL in 100 mg/mL calcium chloride; Villalba Biomedical, Madrid, Spain) and mixed. Mice were anesthetized, and the clotting constructs were injected subcutaneously via a 16 G needle. To characterise the PDCM-CB-P gel, some pieces of the complex were fixed in 2.5% glutaraldehyde for SEM analysis (JeolJSM6330F; Akishima, Tokyo, Japan). After 4 and 12 weeks, mice were sacrificed. Wet weights, volumes, thicknesses, and collagen and sGAG quantities in the constructs were measured.

### Peri-implant bone defect repair in rabbit tibias

Thirsty-six New Zealand rabbits were used for experiments. Rabbits were randomly divided into six groups: PDCM-CB-P, CB-PDCM, PDCM-P, PDCM, HA/TCP, and defect alone groups. The rabbits were anesthetised with ketamine and xylazine. Subsequently, approximately 4 mL bone marrow was aspirated from the posterior iliac crest using a 16 G bone marrow aspiration needle, and the cells were then cultured as described above. PDCM and PRP were prepared as described above.

A functional model was constructed by injecting 500 μL of different group components fabricated with same method described above into the peri-implant bone defects near the epiphyseal line of the rabbit tibia. At 12 weeks after transplantation, rabbits were sacrificed, and samples were prepared for analysis. The rabbits were anesthetised and both tibiae of the rabbits were used for transplantation. Before implant installation, standardised surgical defects were prepared with a 5-mm-wide cylinder-shaped bur. The implants were then placed (micro-arc oxidation surface, length = 5 mm, diameter = 2 mm) and the different group constructs were injected into peri-implant bone defects.

### Statistical analysis

Statistical analyses were performed using SPSS 17.0 software (SPSS, Chicago, IL, USA). Analysis of variance followed by Tukey’s honestly significant difference tests were used for rabbit groups, and paired sample t-tests were used for mouse groups. Results with *P* values less than 0.05 were considered statistically significant.

## Electronic supplementary material

supplementary file

Pressure test of PDCM-CB-P gel

Injection process of PDCM-CB-P gel

Injection process of PDCM-CB-P gel
