# Supplementary material for: Injectable cartilaginous template transformed BMSCs into vascularized bone
Source: Sci Rep. 2018 May 29;8:8244. doi: 10.1038/s41598-018-26472-8 (PMC5973938; doi:10.1038/s41598-018-26472-8)
Supplement: Supplementary file 1 — supplementary file [file 41598_2018_26472_MOESM1_ESM.docx]

**Injectable cartilaginous template transformed BMSCs into vascularized bone**

Xiaoke Feng^1,2#,^, Zhiye Li^1,2#,^, Jianhua Wei^2^, Zhihong Feng^1^, Yimin Zhao^1*^ and Wei Wu^2*^

^1^ State Key Laboratory of Military Stomatology & National Clinical Research Center for Oral Diseases & Shaanxi Key Laboratory of Stomatology, Department of Prosthodontics, School of Stomatology, the Fourth Military Medical University, Xi'an, Shaanxi, 710032, China.

^2^ State Key Laboratory of Military Stomatology & National Clinical Research Center for Oral Diseases & Shaanxi Clinical Research Center for Oral Diseases, Department of Oral & Maxillofacial Surgery, School of Stomatology, the Fourth Military Medical University, Xi'an, Shaanxi, 710032, China.

# These authors contribute equally to this study;

*Corresponding Authors:

Yimin. Zhao, State Key Laboratory of Military Stomatology & National Clinical Research Center for Oral Diseases & Shaanxi Key Laboratory of Stomatology, Department of Prosthodontics, School of Stomatology, the Fourth Military Medical University, Xi'an, Shaanxi, 710032, China.

E-mail: zhaoymdds@126.com

Wei Wu, State Key Laboratory of Military Stomatology & National Clinical Research Center for Oral Diseases & Shaanxi Clinical Research Center for Oral Diseases, Department of Maxillofacial Surgery, School of Stomatology, the Fourth Military Medical University, Xi'an, Shaanxi, 710032, China.

E-mail: wuwdds@163.com

Author contact information:

Xiaoke Feng:

E-mail: 364516576@qq.com

Zhiye Li:

E-mail: 65491397@qq.com

Jianhua Wei:

E-mail: weijhdds@163.com

Zhihong Feng:

E-mail: fengzhihong1520@126.com

**Supplementary method**

1. Mechanical test of PDCM-CB-P gel.

A sample of 500 μL PRP was used for each construct. CBs and PDCMs were collected. For the PDCM-CB-P group, CBs comprised of BMSCs (7.5 × 10^6^ cells) and 1 mL PDCMs were centrifuged into a mixing pellet and resuspended with PRP. We made two models with wax, a 5mm deep cylindrical shape defect, and then we injected the gel into the defects. One minute later, we cut the wax off. The pressure value of PDCM-CB-P gel was determined by the force test instrument (BOSE, Electroforce 3200 seriesⅡ, USA). The sample was fixed into the lower hook with double sticky tape. The force needed to press the sample using a speed of 2 mm/min. All samples were fixed to two identical hooks connected to the load cell and the bottom plate of machine. Pressure was applied to each sample at a rate of 2 mm/min with initial force of 0.00 N until the shape change of samples reached 2.5mm. And the test process has been shooted and uploaded as supplement videos.

**2. Injection property of PDCM-CB-P gel.**

A sample of 1000 μL PRP was used for each construct. CBs and PDCMs were collected. For the PDCM-CB-P group, CBs comprised of BMSCs (1.5 × 10^7^ cells) and 2 mL PDCMs were centrifuged into a mixing pellet and resuspended with PRP. We made two models with wax, a 5mm deep cubical defect and a 5mm deep cylindrical shape defect, and then we injected the gel into the defects. One minute later, we cut the wax off. And the injection process has been shooted and uploaded as supplement videos.

**Supplementary results**

1. **Mechanical test of PDCM-CB-P gel.**


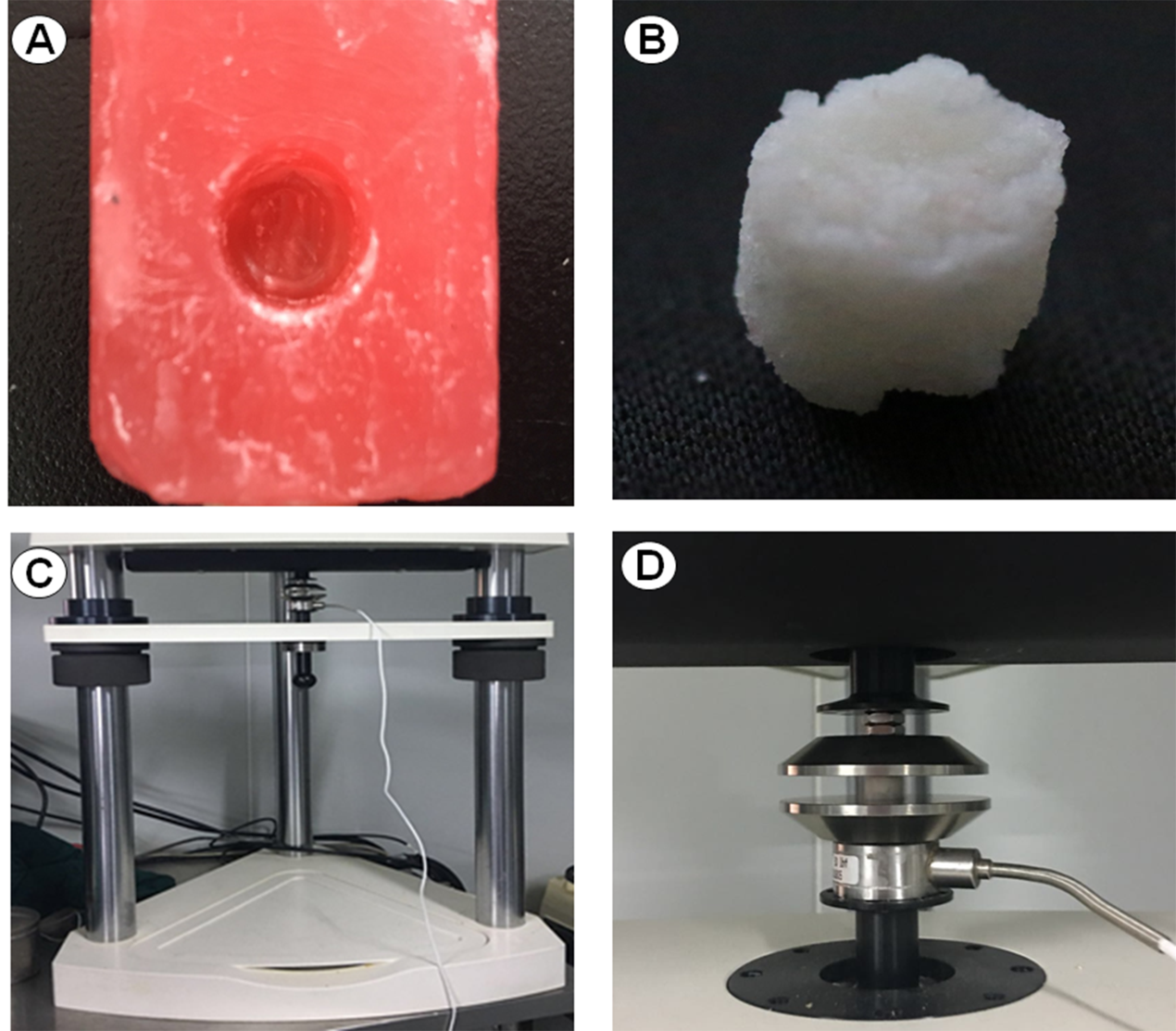


**Supplementary figure 1. Mechanical property of PDCM-CB-P gel.** (A) Cylindrical defect in wax. (B) PDCM-CB-P gel without cylindrical defect wax. (C) The force test instrument. (D) PDCM-CB-P gel’s pressure test.

**Supplementary table 1. Pressure test of** PDCM-CB-P gel

| \| Sample number \| 1 \| 2 \| 3 \| 4 \| 5 \| 6 \| 7 \| 8 \| 9 \| 10 \| Average pressure value(N) \| \| --- \| --- \| --- \| --- \| --- \| --- \| --- \| --- \| --- \| --- \| --- \| --- \| \| Pressure value(N) \| 0.23 \| 0.22 \| 0.24 \| 0.25 \| 0.26 \| 0.24 \| 0.23 \| 0.23 \| 0.25 \| 0.25 \| 0.24 \| |
| --- | --- | --- | --- | --- | --- | --- | --- | --- | --- | --- | --- | --- | --- | --- | --- | --- | --- | --- | --- | --- | --- | --- | --- | --- |
| With the cylindrical defect in wax (supplementary figure 1.A), we got 10 PDCM-CB-P gel samples (supplementary figure 1.B). The pressure test results (supplementary figure 1.C, D, supplementary table 1) showed that the PDCM-CB-P gel can resist an average pressure of 0.25 N. The video of test showed the PDCM-CB-P had not clasped at the end of test (supplementary video 1). |

**2.Injection property of PDCM-CB-P gel.**


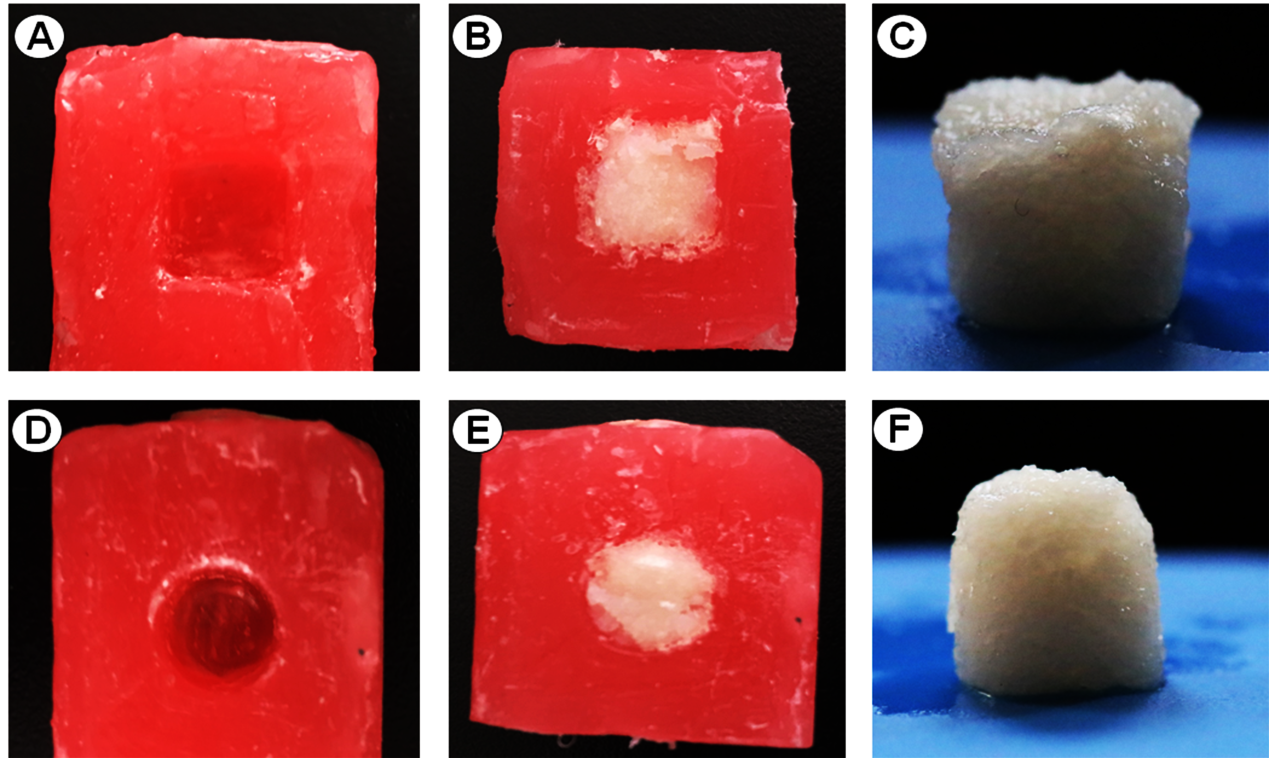


**Supplementary figure 2. Injection property of PDCM-CB-P gel.** (A) Cubical defect in wax. (B) Cubical defect filled with PDCM-CB-P gel. (C) PDCM-CB-P gel without cubical defect wax. (D) Cylindrical defect in wax. (E) Cylindrical defect filled with PDCM-CB-P gel. (F) PDCM-CB-P gel without cylindrical defect wax.

As the supplementary video 2, 3 showed the prepared PDCM-CB-P gel could be injected smoothly though the 16G needle. We made two models with wax, the cubical defect and cylindrical shape defect (supplement figure.1B, E), and then injected the gel into the defects (supplement figure.1C, F). One minute later, we cut the wax off and found the gel had the same shape as the defects as the supplement figure.1C, F show.
